# Energy In-Equivalence in Australian Marsupials: Evidence for Disruption of the Continent’s Mammal Assemblage, or Are Rules Meant to Be Broken?

**DOI:** 10.1371/journal.pone.0057449

**Published:** 2013-02-27

**Authors:** Adam J. Munn, Craig Dunne, Dennis W. H. Müller, Marcus Clauss

**Affiliations:** 1 Institute of Conservation Biology and Environmental Management, School of Biological Science, University of Wollongong, Wollongong, Australia; 2 Clinic for Zoo Animals, Exotic Pets and Wildlife, Vetsuisse Faculty, University of Zurich, Zurich, Switzerland; University of Sydney, Australia

## Abstract

The energy equivalence rule (EER) is a macroecological hypothesis that posits that total population energy use (PEU) should be independent of species body mass, because population densities and energy metabolisms scale with body mass in a directly inverse manner. However, evidence supporting the EER is equivocal, and the use of basal metabolic rate (BMR) in such studies has been questioned; ecologically-relevant indices like field metabolic rate (FMR) are probably more appropriate. In this regard, Australian marsupials present a novel test for the EER because, unlike eutherians, marsupial BMRs and FMRs scale differently with body mass. Based on either FMR or BMR, Australian marsupial PEU did not obey an EER, and scaled positively with body mass based on ordinary least squares (OLS) regressions. Importantly, the scaling of marsupial population density with body mass had a slope of −0.37, significantly shallower than the expected slope of −0.75, and not directly inverse of body-mass scaling exponents for BMR (0.72) or FMR (0.62). The findings suggest that the EER may not be a causal, universal rule, or that for reasons not yet clear, it is not operating for Australia’s unique native fauna.

## Introduction

Identifying mechanistic associations between organism body size (mass), resource use and whole-ecosystem processes is central to predicting how different species and their ecosystems might respond to environmental challenges, or to other factors affecting body size and resource use (e.g. [1]). One theory that considers how organism size and resource needs affect abundance, and ultimately whole ecosystems processes, is the Energy Equivalence Rule (EER; [2], [3], [4]; also described as the Energetic Equivalence Rule). In short, the EER is a type of size-density relationship that states that total population energy-fluxes by different species should be equivalent, regardless of their respective body masses [2], [3], [5], [6]. The EER has been used to explain a range of large-scale ecological phenomena, from community structuring to global biodiversity patterns (see [5], [6], [7], [8]), but the validity of some of its underlying features have been questioned, and evidence supporting the idea as a general ‘rule’ is equivocal [2], .

The EER was derived from the observation that individual energy requirements (or metabolic rate; kJ d^−1^) apparently scale with animal body mass raised to a power close to 0.75 (i.e. mass^0.75^), whereas the scaling of animal population densities (individuals km^−2^) scale with body mass raised to a power close to −0.75 (i.e. mass^−0.75^; [15], i.e. the direct inverse of metabolic rate [2], [3]). Consequently, the EER states that whole-population energy fluxes (kJ per unit area) should be the same for differently-sized organisms, because total population energy use (PEU) equals energy turnover (or basal metabolic rate; BMR) multiplied by its density; i.e. [BMR•mass^0.75^] • [Density•mass^−0.75^] = PEU•mass^0^ [2], [3]. Leaving aside debate concerning the ‘correct’ scaling exponent for BMR ( [8], [15], [16], [17]; see also McNab’s MISTCHEF model for the scaling of metabolism in mammals [18]), an EER should possess ecological relevance provided the pertinent scaling exponents for energy metabolism and density are inversely related [5].

There is empirical support for [2], [3], [4], [12], [19], [20] and against [4], [6], [9], [12], [21] the EER. One concern is whether BMR is appropriate for deriving PEU, and that EERs should focus on ecologically-relevant indices like field metabolic rate (FMR; [11]); FMRs are typically 2–3 times BMR for mammals [15]. Axiomatically, using BMR may not be problematic provided it scales with the same slope as does FMR (e.g. mass^0.75^), which is apparently the case for eutherian mammals [15]. However, comparable scaling of BMR and FMR is not apparent for all mammal groups [15], and one notable exception includes the marsupial fauna of continental Australia.

Australian marsupial BMRs scale with a body-mass exponent of 0.72 [15], [22], but their FMRs scale with a body-mass exponent of 0.62 ( [15]; this study). Therefore, Australian marsupials present a novel group for testing the EER, partly because of their divergent body-mass scaling exponents for BMR and FMR, and also because they have largely evolved isolated from the eutherian ecological-analogues on other continents.

## Materials and Methods

Density data were collected from published studies [23], [24] for n = 68 species of Australian marsupial, spanning three orders of magnitude of body mass (Table S1) that encompassed the full spectrum of extant marsupial sizes. Data for marsupial BMRs were collated for n = 52 species, and FMRs collated for n = 37 species (Table S2 and Table S3). Specifically for FMR, we collated data for adult, non-reproductive (i.e. non-lactating/non-pregnant) animals covering three orders of magnitude (Table S3). When more than one measure of FMR was available for a species (e.g. seasonal studies) we used minimal values, usually representing dry season data (FMRs in other seasons or following rainfall are typically higher). By excluding data on juvenile (still growing) or lactating animals we present the most conservative dataset for marsupial FMRs to date, with the view to present the minimum free-living resource requirements of Australian marsupials generally.

We explored the scaling of marsupial population densities, BMRs, FMRs and PEUs against body mass using ordinary least squares (OLS) regressions on log_10_-transformmed data (normality of respective residuals was tested using Shaprio-Wilks test). Log_10_-PEU was derived by multiplying raw BMR and FMR by density prior to log transformation. Not all species for which we had compiled density information (n = 68) were represented in the BMR (n = 52) or FMR (n = 37) datasets. Therefore, to test whether scaling patterns in the species subsets for BMR OR FMR were representative of whole datasets, we analysed whether the interaction of body mass and the presence or absence of data-overlap was statistically significant, using general linear models. A non-significant interaction with body mass indicated that the regression slopes were not significantly different, and were hence representative of slopes derived from each entire dataset. However, for the FMR dataset there were only two species for which estimates of density were not available, making formal statistical comparisons impossible; we therefore assumed that the slopes of the FMR_Density_ data-subset was representative of marsupials generally. Slopes derived were further compared with predicted slopes where appropriate using Z-tests.

## Results

Marsupial density (entire dataset) scaled with body mass with an exponent of −0.37, and was significantly different from a slope of −0.75 (Table 1; Z = 6.1, *P*<0.0001). Importantly, the slopes for density-scaling regressions for marsupials that included data on BMR or FMR were not significantly different from the entire dataset (Density_BMR_ interaction F_1, 64_ = 0.004, *P* = 0.95; Density_FMR_ interaction F_1, 64_ = 0.001, *P = *0.98).

**Table 1:** Scaling (OLS regressions on log_10_-transformed data) of Australian marsupial population density (number of individuals km^−2^), basal (BMR) and field (FMR) metabolic rate (kJ d^−1^) with body mass (g).

Parameter	a	*b*	*R* ^2^	*P*
Density (n = 68)	3.19±0.20 (2.79–3.59)	−0.37±0.06 (−0.50 to −0.25)	0.394	**<0.0001**
BMR (n = 52)	0.169±0.051 (0.067–0.271 )	0.72±0.02 (0.69–0.76)	0.967	**<0.0001**
FMR (n = 37)	0.877±0.057 (0.761–0.993)	0.62±0.018 (0.59–0.66)	0.972	**<0.0001**
**BMR Overlap (n = 36)**				
Density_BMR_	3.25±0.30 (2.63–3.86)	−0.39±0.1 (−0.60 to −0.19)	0.313	**<0.0001**
BMR_Density_	0.320±0.074 (0.169–0.471)	0.68±0.025 (0.63–0.73)	0.956	**<0.0001**
**FMR Overlap (n = 35)**				
Density_FMR_	3.22±0.31 (2.60–3.84)	−0.38±0.09 (−0.57 to −0.19)	0.328	**<0.0001**
FMR_Density_	0.889±0.059 (0.77–1.01)	0.62±0.02 (0.58–0.66)	0.972	**<0.0001**

Regressions were performed separately for all species for which density, BMR and FMR were available, in addition to those for which overlapping data were available (Note: overlap data for density, BMR and FMR are identified by respective subscripts, e.g. Density_BMR_ = density data for which BMR is also available; values in parentheses are 95% confidence limit ranges).

**Figure 1:**
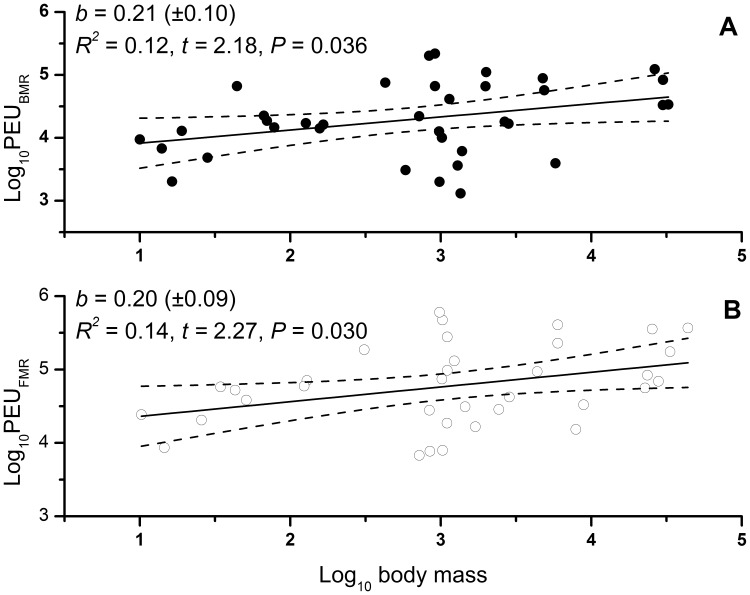
Scaling (OLS regressions on log_10_-transformed data) of Australian marsupial population energy use (PEU; kJ d^−1^ km^−2^) with body mass, based on (A) basal metabolic rate (BMR; kJ d^−1^) and (B) field metabolic rate (FMR; kJ d^−1^).

Marsupial BMR (entire dataset) scaled with a body mass exponent of 0.72 (Table 1). The slopes for BMR-scaling for marsupials with and without density data were not significantly different (BMR_Density_ interaction F_1, 49_ = 2.1, *P* = 0.16; Table 1). Marsupial FMR (entire dataset) scaled with body mass with an exponent of 0.62 (Table 1). Notably, the 95% confidence intervals for the exponents of either of the BMR or FMR did not include the reciprocal of the 95% confidence interval for the density scaling exponent; in other words, the scaling exponents of neither BMR nor FMR were directly inverse that for population density (Table 1). Consequently, marsupial population energy use based on measures of BMR (PEU_BMR_) scaled with a positive body-mass exponent of 0.21 (*P* = 0.036; Fig. 1). Similarly, marsupial population energy use based on measures of FMR (PEU_FMR_) scaled with a body mass exponent of 0.20 (*P* = 0.030; Fig. 1).

## Discussion

Australian marsupials do not follow an EER according to the OLS-regressions of PEU based on either BMR or FMR (Table 1). Most importantly, a key feature of the EER is that the body-mass scaling exponents for density and energy turnover (BMR or FMR) should be a direct inverse of one another, yet for both BMR and FMR the scaling exponents did not demonstrate a reciprocal overlap with the scaling exponent for population density (see also [23]). We propose two alternative hypotheses to explain why Australian marsupials do not obey an EER. Firstly, and perhaps most parsimoniously, the EER may not be a general ecological ‘rule’, and as such it is not universal or predictive. Alternatively, the EER may indeed be causal, but for reasons that are not yet clear it is not operating at the continental scale for Australian marsupials. There is evidence to support both of these lines of argument.

That the EER formulated by Damuth [2],[3] is not in fact a ‘rule’ is somewhat supported by our data, and is comparable with other studies that refute EERs across a range of species, communities and trophic levels (e.g. [6]). As such, global-scale EERs (sensu White et al. [5]) may be emergent artefacts that are not be driven by bottom-up, local EERs (see also [6],[15]), but firm conclusions would require that all sympatric species be included in any analyses. Furthermore, it is worth noting that the composition of Australia’s extant marsupial fauna comprises of mainly small carnivores/omnivores, but medium-large herbivores (mainly the wombats and kangaroos). It is well-known that diet influences BMR and FMR (e.g. see [15], [18], [25], [26] and references therein). Nonetheless, local- and taxon-scale species-density patterns are rarely independent of body size [5], [6], [14], [27], but the mechanisms explaining such outcomes are unclear. Furthermore, there is considerable heterogeneity in the scaling exponent of animal BMRs at least, with around 50% of orders displaying slopes for body-mass: BMR scaling that deviate from the expected (according to EER) slope 0.75 ( [8], see also [28], [29]). Therefore, it appears unrealistic to assume that global-EERs based on a single body-mass: density scaling exponent (e.g. 0.75) should apply locally. Instead, EERs should be tailored to the species-specific energy requirements and interactions for all species in a given area. Consequently, outright dismissal of an EER for Australian marsupials may be premature because we could not include information for all sympatric species, and particularly for the numerous introduced domestic and feral eutherians that have helped to transform Australia’s biomes since their introductions with Europeans some 200 years ago.

Dramatic changes to Australia’s landscapes since European arrival have precipitated major declines, even to extinction, of numerous small-medium sized marsupials, along with increases in the population sizes of the largest extant marsupials, particularly the grazing kangaroos [30]. Widespread land clearing and the establishment of permanent water sources for grazing ruminants, mainly sheep and cattle, have contributed to the declines of Australia’s small-medium sized marsupials, in addition to supporting the proliferation of some larger, grazing marsupials such as kangaroos [30], [31]. Additionally, there has been widespread control and exclusion of Australia’s largest established predators, the mainland dingo/wild dog. This has apparently released some larger marsupial species from predation pressures (e.g. kangaroos), whilst concomitantly enabling introduced meso-predators (foxes and cats) to target small-medium marsupials, possibly driving down their population numbers or restricting them to sub-optimal refuges where their abundances are lower than might expected without such heavy predation pressures [32], [33], [34]. Moreover, the control of large predators and the spread of grasslands and artificial water sources (e.g. dams, tanks, bores) have supported extensive infiltrations of introduced herbivores like rabbits, goats and camel, as well as omnivores like pigs, which have further transformed Australia’s biomes and species compositions. Therefore, it is not wholly unexpected that the scaling of Australian marsupial density: body mass might differ from that of mammals generally, and particularly from that of eutherians on other continents [2], [3], [6].

Eutherians generally have higher energy requirements than marsupials [15], [22], and the introduction of eutherians to Australia in high numbers as free-ranging domestic stock, and as extensive feral populations, could act to counter-balance, or even over-balance energy fluxes through Australian ecosystems. As such, Australia presents a unique opportunity to test EERs along gradients of disturbance at local, regional and continental scales. Unfortunately, there are presently too few data on the FMRs of Australian native and non-native eutherians to adequately compare the contributions of Australia’s marsupials with that of native and introduced eutherians to total-ecosystem energy fluxes.

Importantly, Australia’s extant marsupials are not representative of the continents’ pre-European assemblages, and numerous small-medium sized marsupials are now extinct [35], [36]. Consequently, evaluating the role of phylogeny in the PEU patterns for Australian marsupials is complicated by the likely influence of phylogeny (and body mass) on the extinction-risk of Australia’s small-medium sized marsupials over the last 200 years, which may or may not be cross-correlated with their metabolic physiology (for further discussion on the risks of misinterpreting phylogenetic influences see [18], [37]). For example, torpor and hibernation have apparently mitigated extinction risks for many small mammals, but larger mammals that maintain homoeothermic body temperatures may suffer higher extinction rates, presumably because of their need to sustain high and relatively constant energy metabolism [38]; although to some degree large animals may ameliorate risks via migration to avoid climatic or other pressures. Phylogeny is therefore important, as has been demonstrated for mammalian BMRs across a range of taxa including marsupials [25], [28], but other factors likely contribute to the patterns we observe [18], [25], [28]. Indeed, White et al. [29] has recommended that more parameter-rich models (that include phylogeny) are needed to fully appreciate the ecological patterns associated with mammalian BMR: body mass allometries, and we suggest that these ideas ought to extend to include FMR because that is the physiological level at which species operate ecologically [11]. Nonetheless, the specific hypothesis that we are testing here (that the EER holds true for extant Australian marsupials) does not directly concern phylogeny in that we are interested in present-day ecological patterns, rather than the evolution of energy metabolism or population density/energy use per se. Perhaps after sufficient data are collected for the energy metabolisms and population densities of Australia’s non-marsupial mammals, and indeed birds and reptiles, might we fully appreciate the extent to which phylogeny contributes to the macroecology of population-energy fluxes for Australia’s extant fauna and their ecosystems. Regardless, our continental-wide examination of marsupial density: body mass scaling reveals that Australia’s extant marsupials may be experiencing profound ecological imbalances, the consequences of which are probably still unfolding.

## Acknowledgments

Sincere thanks to Professor Kris French, Dr Phil Byrne and Dr Terry O’Dwyer for thoughtful discussions and feedback on earlier drafts of this manuscript. Thanks also to Professors Chris Johnson and Don Bradshaw for providing raw data to assist with our collation of density and FMRs, along with Professor Mark Westoby and Dr Matt Symonds for discussion on regressions and phylogeny, and to Dr Matt Symonds and two anonymous reviewers for their comments and constructive criticism of earlier versions of this manuscript.

## Supporting Information

Table S1 **Australian marsupial population density (individual km^−2^) and body mass (g).**
(DOCX)

Table S2 **Australian marsupial basal metabolic rate (BMR) and body mass (g).**
(DOCX)

Table S3 **Australian marsupial field metabolic rate (FMR) and body mass (g).**
(DOCX)
