# Supplementary material for: Energy In-Equivalence in Australian Marsupials: Evidence for Disruption of the Continent’s Mammal Assemblage, or Are Rules Meant to Be Broken?
Source: PLoS One. 2013 Feb 27;8(2):e57449. doi: 10.1371/journal.pone.0057449 (PMC3583869; doi:10.1371/journal.pone.0057449)
Supplement: Table S1 — Australian marsupial population density (individual km−2) and body mass (g). (DOCX) [file pone.0057449.s001.docx]

Table S1: Australian marsupial population density (individual km^-2^) and body mass (g).

| **Genus species** | **Density**  **(individuals km^-2^)** | **Body mass**  **(g)** | **Reference** |
| --- | --- | --- | --- |
| *Acrobates pygmaeus* | 900 | 12.5 | [[1](#_ENREF_1)] |
| *Aepyprimnus rufescens* | 31 | 2750 | [[1](#_ENREF_1)] |
| *Antechinus agilis* | 711 | 24 | [[1](#_ENREF_1)] |
| *Antechinus bellus* | 160 | 44.5 | [[1](#_ENREF_1)] |
| *Antechinus favipes* | 730 | 45 | [[1](#_ENREF_1)] |
| *Antechinus leo* | 162 | 74 | [[1](#_ENREF_1)] |
| *Antechinus minimus maritimus* | 800 | 61.3 | [[1](#_ENREF_1)] |
| *Antechinus stuartii* | 285 | 27.5 | [[1](#_ENREF_1)] |
| *Antechinus swainsonii* | 709 | 53 | [[1](#_ENREF_1)] |
| *Betongia penicillata* | 36 | 1270 | [[1](#_ENREF_1)] |
| *Bettongia gaimardi* | 19 | 1660 | [[1](#_ENREF_1)] |
| *Bettongia lesueur* | 16 | 1280 | [[1](#_ENREF_1)] |
| *Burramys parvus* | 1800 | 41.5 | [[1](#_ENREF_1)] |
| *Cercartetus nanus* | 311 | 24 | [[1](#_ENREF_1)] |
| *Dasyursus viverrinus* | 10 | 1100 | [[1](#_ENREF_1)] |
| *Dasyurus geoffroyi* | 2 | 1100 | [[1](#_ENREF_1)] |
| *Dasyurus hallacatus* | 21 | 634 | [[1](#_ENREF_1)] |
| *Dendrolagus bennettianus* | 28 | 10525 | [[1](#_ENREF_1)] |
| *Dendrolagus lumholtzi* | 145 | 7025 | [[1](#_ENREF_1)] |
| *Gymnobelideus leadbeateri* | 313 | 127.5 | [[1](#_ENREF_1)] |
| *Hemibelideus lemuroides* | 700 | 974.5 | [[1](#_ENREF_1)] |
| *Hypsiprymnodon moschatus* | 240 | 520 | [[1](#_ENREF_1)] |
| *Isoodon auratus* | 1000 | 485 | [[1](#_ENREF_1)] |
| *Isoodon obesulus* | 197 | 755 | [[1](#_ENREF_1)] |
| *Lagorchestes conspicillatus* | 42 | 3175 | [[1](#_ENREF_1)] |
| *Lagorchestus hirsutus* | 47 | 1265 | [[1](#_ENREF_1)] |
| *Lasiorhinus krefftii* | 22 | 31450 | [[1](#_ENREF_1)] |
| *Lasiorhinus latifrons* | 22 | 27125 | [[1](#_ENREF_1)] |
| *Macropus agilis* | 18 | 15000 | [[1](#_ENREF_1)] |
| *Macropus antilopinus* | 75 | 27025 | [[1](#_ENREF_1)] |
| *Macropus eugenii* | 82 | 6500 | [[1](#_ENREF_1)] |
| *Macropus fuliginosus* | 57 | 36500 | [[1](#_ENREF_1)] |
| *Macropus giganteus* | 45 | 40750 | [[1](#_ENREF_1)] |
| *Macropus parryi* | 39 | 13500 | [[1](#_ENREF_1)] |
| *Macropus robustus* | 31 | 25375 | [[1](#_ENREF_1)] |
| *Macropus rufogriseus* | 48 | 16200 | [[1](#_ENREF_1)] |
| *Macropus rufus* | 12 | 40000 | [[1](#_ENREF_1)] |
| *Macrotis lagotis* | 16 | 1350 | [[1](#_ENREF_1)] |
| *Onychogalea fraenata* | 20 | 5750 | [[1](#_ENREF_1)] |
| *Parantechinus apicalis* | 600 | 74.5 | [[1](#_ENREF_1)] |
| *Perameles gunni* | 480 | 870 | [[1](#_ENREF_1)] |
| *Petauroides volans* | 143 | 1300 | [[1](#_ENREF_1)] |
| *Petaurus australis* | 15 | 557.5 | [[1](#_ENREF_1)] |
| *Petaurus breviceps* | 377 | 127.5 | [[1](#_ENREF_1)] |
| *Petaurus norfolcensis* | 211 | 230 | [[1](#_ENREF_1)] |
| *Petrogale assimilis* | 125 | 4500 | [[1](#_ENREF_1)] |
| *Petrogale lateralis* | 275 | 4000 | [[1](#_ENREF_1)] |
| *Petrogale xanthopus* | 15 | 8500 | [[1](#_ENREF_1)] |
| *Phascogale tapoatafa* | 111 | 193.5 | [[1](#_ENREF_1)] |
| *Phascolarctos cinereus* | 170 | 8025 | [[1](#_ENREF_1)] |
| *Potorous longipes* | 32 | 1900 | [[1](#_ENREF_1)] |
| *Potorous tridactylus* | 60 | 1100 | [[1](#_ENREF_1)] |
| *Pseudocheirus herbertensis* | 625 | 1110.5 | [[1](#_ENREF_1)] |
| *Pseudocheirus occidentalis* | 302 | 987.5 | [[1](#_ENREF_1)] |
| *Pseudocheirus peregrinus* | 1003 | 800 | [[1](#_ENREF_1)] |
| *Sarcophilus harrisii* | 6 | 9000 | [[1](#_ENREF_1)] |
| *Sminthopsis crassicaudata* | 160 | 15 | [[1](#_ENREF_1)] |
| *Sminthopsis leucopus* | 618 | 24 | [[1](#_ENREF_1)] |
| *Sminthopsis murina* | 600 | 17 | [[1](#_ENREF_1)] |
| *Tarsipes rostratus* | 645 | 10.5 | [[2](#_ENREF_2)] |
| *Thylogale billardierii* | 250 | 5450 | [[1](#_ENREF_1)] |
| *Thylogale thetis* | 360 | 5400 | [[1](#_ENREF_1)] |
| *Trichosurus arnhemensis* | 300 | 2625 | [[1](#_ENREF_1)] |
| *Trichosurus caninus* | 66 | 3500 | [[1](#_ENREF_1)] |
| *Trichosurus vulpecula* | 210 | 2625 | [[1](#_ENREF_1)] |
| *Vombatus ursinus* | 34 | 26000 | [[1](#_ENREF_1)] |
| *Wallabia bicolor* | 31 | 15000 | [[1](#_ENREF_1)] |
| *Wyulda squamicaudata* | 345 | 1450 | [[1](#_ENREF_1)] |

**Table S1 References**

1. Fisher DO, Owens IPF, Johnson CN (2001) The ecological basis of life history variation in marsupials. Ecology 82: 3531-3540.

2. Bradshaw SD, Phillips RD, Tomlinson S, Holley RJ, Jennings S, et al. (2007) Ecology of the Honey possum, *Tarsipes rostratus*, in Scott National Park, Western Australia. Australian Mammalogy 29: 25-38.
