# Supplementary material for: Energy In-Equivalence in Australian Marsupials: Evidence for Disruption of the Continent’s Mammal Assemblage, or Are Rules Meant to Be Broken?
Source: PLoS One. 2013 Feb 27;8(2):e57449. doi: 10.1371/journal.pone.0057449 (PMC3583869; doi:10.1371/journal.pone.0057449)
Supplement: Table S2 — Australian marsupial basal metabolic rate (BMR) and body mass (g). (DOCX) [file pone.0057449.s002.docx]

Table S2: Australian marsupial basal metabolic rate (BMR) and body mass (g).

| **Genus species** | **BMR**  **(kJ d^-1^)** | **Body mass**  **(g)** | **Reference** |
| --- | --- | --- | --- |
| *Acrobates pygmaeus* | 7.5 | 14.0 | [[1](#_ENREF_1),[2](#_ENREF_2)] |
| *Aepyprimnus rufescens* | 540 | 2820 | [[1](#_ENREF_1)] |
| *Antechinomys laniger* | 12.7 | 25.8 | [[1](#_ENREF_1)] |
| *Antechinus stuartii* | 17.0 | 28.2 | [[1](#_ENREF_1)] |
| *Antechinus swainsonii* | 31.8 | 66.9 | [[1](#_ENREF_1)] |
| *Betongia penicillata* | 283 | 1018 | [[1](#_ENREF_1)] |
| *Bettongia gaimardi* | 323 | 1385 | [[1](#_ENREF_1)] |
| *Burramys parvus* | 36.8 | 44.3 | [[1](#_ENREF_1)] |
| *Cercartetus conncinnus* | 11.2 | 18.6 | [[1](#_ENREF_1)] |
| *Cercartetus lepidus* | 9.5 | 12.6 | [[1](#_ENREF_1)] |
| *Cercartetus nanus* | 60.2 | 70.0 | [[1](#_ENREF_1)] |
| *Dasycercus cristicauda* | 25.7 | 101 | [[1](#_ENREF_1)] |
| *^#^Dasyuroides byrnei* | 36.1 | 91.7 | [[1](#_ENREF_1)] |
| *Dasyursus viverrinus* | 200 | 982 | [[1](#_ENREF_1)] |
| *Dasyurus geoffroyi* | 569 | 1354 | [[1](#_ENREF_1)] |
| *Dasyurus hallacatus* | 150 | 584 | [[1](#_ENREF_1),[2](#_ENREF_2),[3](#_ENREF_3)] |
| *Dasyurus maculatus* | 281 | 1782 | [[1](#_ENREF_1)] |
| *Gymnobelideus leadbeateri* | 51.9 | 166 | [[4](#_ENREF_4)] |
| *Isoodon auratus* | 75.5 | 428 | [[1](#_ENREF_1)] |
| *Isoodon macrourus* | 289 | 1551 | [[1](#_ENREF_1)] |
| *Isoodon obesulus* | 112 | 717 | [[1](#_ENREF_1)] |
| *Lagorchestes conspicillatus* | 429 | 2660 | [[4](#_ENREF_4)] |
| *Lasiorhinus latifrons* | 1508 | 29917 | [[1](#_ENREF_1)] |
| *Macropus eugenii* | 701 | 4878 | [[4](#_ENREF_4)] |
| *Macropus giganteus* | 2737 | 26400 | [[1](#_ENREF_1),[5](#_ENREF_5)] |
| *Macropus robustus* | 2691 | 30000 | [[1](#_ENREF_1),[2](#_ENREF_2)] |
| *Macropus rufus* | 2947 | 32490 | [[4](#_ENREF_4),[5](#_ENREF_5)] |
| *Macrotis lagotis* | 227 | 1294 | [[1](#_ENREF_1)] |
| *Myrmecobius fasciatus* | 71.8 | 400 | [[1](#_ENREF_1)] |
| *Ningaui yvonnae* | 7.9 | 11.6 | [[1](#_ENREF_1)] |
| *Parantechinus apicalis* | 24.4 | 78.3 | [[6](#_ENREF_6)] |
| *Perameles gunni* | 420 | 837 | [[1](#_ENREF_1)] |
| *Perameles nasuta* | 159 | 645 | [[1](#_ENREF_1)] |
| *Petauroides volans* | 289 | 1141 | [[1](#_ENREF_1)] |
| *Petaurus breviceps* | 45.3 | 127 | [[1](#_ENREF_1)] |
| *Phascogale tapoatafa* | 127 | 157 | [[1](#_ENREF_1)] |
| *Phascolarctos cinereus* | 521 | 4765 | [[1](#_ENREF_1)] |
| *Planigale gilesi* | 3.5 | 9.1 | [[1](#_ENREF_1)] |
| *Planigale ingrami* | 5.7 | 7.1 | [[4](#_ENREF_4)] |
| *Planigale maculata* | 6.0 | 10.8 | [[1](#_ENREF_1)] |
| *Planigale tenuirostris* | 5.7 | 7.1 | [[1](#_ENREF_1)] |
| *Potorous tridactylus* | 210 | 976 | [[1](#_ENREF_1)] |
| *Pseudocheirus occidentalis* | 219 | 917 | [[1](#_ENREF_1),[2](#_ENREF_2)] |
| *Pseudocheirus peregrinus* | 217 | 916 | [[1](#_ENREF_1)] |
| *Sarcophilus harrisii* | 668 | 5775 | [[1](#_ENREF_1)] |
| *Setonix brachyurus* | 420 | 2674 | [[4](#_ENREF_4)] |
| *Sminthopsis crassicaudata* | 12.7 | 16.4 | [[1](#_ENREF_1)] |
| *Sminthopsis macroura* | 11.4 | 19.4 | [[1](#_ENREF_1)] |
| *Sminthopsis murina* | 21.5 | 19.0 | [[1](#_ENREF_1)] |
| *Spilocuscus maculatus* | 576 | 4250 | [[1](#_ENREF_1)] |
| *Tarsipes rostratus* | 14.6 | 10.0 | [[1](#_ENREF_1)] |
| *Trichosurus arnhemensis* | 369 | 2005 | [[1](#_ENREF_1)] |
| *Trichosurus vulpecula* | 315 | 1982 | [[1](#_ENREF_1),[2](#_ENREF_2)] |

^#^also reported as *Dasycercus byrnie*.

**Table S2 References**

1. White CR, Phillips NF, Seymour RS (2006) The scaling and temperature dependence of vertebrate metabolism. Biology Letters 2: 125-127.

2. Hume ID (1999) Marsupial Nutrition. Cambridge: Cambridge University Press.

3. Withers PC, Thompson GG, Seymour RS (2000) Metabolic physiology of the north-western marsupial mole, *Notoryctes caurinus* (Marsupialia : Notoryctidae). Australian Journal of Zoology 48: 241-258.

4. White CR, Seymour RS (2003) Mammalian basal metabolic rate is proportional to body mass^2/3^. Proceedings of the National Academy of Sciences 100: 4046-4049.

5. Dawson TJ, Blaney CE, Munn AJ, Krockenberger A, Maloney SK (2000) Thermoregulation by kangaroos from mesic and arid habitats: Influence of temperature on routes of heat loss in eastern grey kangaroos (*Macropus giganteus*) and red kangaroos (*Macropus rufus*). Physiological and Biochemical Zoology 73: 374-381.

6. Withers PC, Cooper CE (2011) Using a priori contrasts for multivariate repeated-measures ANOVA to analyze thermoregulatory responses of the Dibbler (*Parantechinus apicalis*; Marsupialia, Dasyuridae). Physiological and Biochemical Zoology 84: 514-521.
