# Supplementary material for: Energy In-Equivalence in Australian Marsupials: Evidence for Disruption of the Continent’s Mammal Assemblage, or Are Rules Meant to Be Broken?
Source: PLoS One. 2013 Feb 27;8(2):e57449. doi: 10.1371/journal.pone.0057449 (PMC3583869; doi:10.1371/journal.pone.0057449)
Supplement: Table S3 — Australian marsupial field metabolic rate (FMR) and body mass (g). (DOCX) [file pone.0057449.s003.docx]

Table S3: Australian marsupial field metabolic rate (FMR) and body mass (g).

| **Genus species** | **FMR**  **(kJ d^-1^)** | **Body mass**  **(g)** | **Reference** |
| --- | --- | --- | --- |
| *Aepyprimnus rufescens* | 1362 | 2860 | [[1](#_ENREF_1)] |
| *Antechinus minimus maritimus* | 72.4 | 34.4 | [[2](#_ENREF_2)] |
| *Antechinus stuartii* | 72.0 | 25.7 | [[3](#_ENREF_3)] |
| *Antechinus swainsonii* | 74.0 | 43.0 | [[4](#_ENREF_4)] |
| *Betongia penicillata* | 524 | 1100 | [[4](#_ENREF_4)] |
| *Bettongia gaimardi* | 874 | 1700 | [[4](#_ENREF_4)] |
| *Bettongia lesueur* | 422 | 720 | [[5](#_ENREF_5)] |
| *Dasyursus viverrinus* | 793 | 1029 | [[3](#_ENREF_3)] |
| *Gymnobelideus leadbeateri* | 226 | 129 | [[3](#_ENREF_3),[6](#_ENREF_6)] |
| *Hemibelideus lemuroides* | 675 | 1026 | [[3](#_ENREF_3)] |
| *Isoodon auratus* | 186 | 310 | [[7](#_ENREF_7)] |
| *Isoodon obesulus* | 667 | 1231 | [[8](#_ENREF_8)] |
| *Lagorchestes conspicillatus* | 680 | 2430 | [[5](#_ENREF_5)] |
| *Lagorchestus hirsutus* | 661 | 1453 | [[4](#_ENREF_4)] |
| *Lasiorhinus krefftii* | 3802 | 23625 | [[9](#_ENREF_9)] |
| *Lasiorhinus latifrons* | 3142 | 28000 | [[9](#_ENREF_9)] |
| *Macropus eugenii* | 1150 | 4380 | [[10](#_ENREF_10)] |
| *Macropus fuliginosus* | 6245 | 25383 | Munn et al., unpublished |
| *Macropus giganteus* | 8170 | 43900 | [[11](#_ENREF_11)] |
| *Macropus rufus* | 4872 | 22750 | [[12](#_ENREF_12)] |
| *Macrotis lagotis* | 480 | 848 | [[3](#_ENREF_3)] |
| *Parantechinus apicalis* | 64.0 | 51.0 | [[4](#_ENREF_4)] |
| *Petauroides volans* | 520 | 1018 | [[13](#_ENREF_13)] |
| *Petaurus breviceps* | 158 | 124 | [[14](#_ENREF_14)] |
| *Petrogale xanthopus* | 2209 | 8900 | [[4](#_ENREF_4)] |
| *Phascogale calura* | 54.0 | 30.0 | [[15](#_ENREF_15)] |
| *Phascolarctos cinereus* | 1350 | 5980 | [[16](#_ENREF_16),[17](#_ENREF_17)] |
| *Potorous tridactylus* | 463 | 839 | [[3](#_ENREF_3)] |
| *Pseudocheirus herbertensis* | 446 | 1103 | [[3](#_ENREF_3)] |
| *Pseudocheirus peregrinus* | 602 | 981 | [[18](#_ENREF_18)] |
| *Sarcophilus harrisii* | 2591 | 7900 | [[4](#_ENREF_4)] |
| *Setonix brachyurus* | 548 | 1900 | [[3](#_ENREF_3),[10](#_ENREF_10)] |
| *Sminthopsis crassicaudata* | 53.9 | 14.5 | [[3](#_ENREF_3),[19](#_ENREF_19)] |
| *Tarsipes rostratus* | 37.6 | 10.3 | [[3](#_ENREF_3),[20](#_ENREF_20)] |
| *Thylogale billardierii* | 1630 | 5980 | [[3](#_ENREF_3),[11](#_ENREF_11)] |
| *Trichosurus arnhemensis* | 324 | 1103 | [[5](#_ENREF_5)] |
| *Vombatus ursinus* | 5139 | 33388 | [[9](#_ENREF_9)] |

**Table S3 References**

1. Wallis I, Green B (1992) Seasonal field energetics of the rufous rat-kangaroo (*Aepyprymnus rufescens*). Australian Journal of Zoology 40: 279-290.

2. Sale MG, Speakman JR, Arnould JPY (2009) Energy expenditure, water flux, and activity budgets of female swamp Antechinuses in contrasting habitats. Journal of Mammalogy 90: 1238-1245.

3. Nagy KA, Girard IA, Brown TK (1999) Energetics of free-ranging mammals, reptiles, and birds. Annual Review of Nutrition 19: 247-277.

4. Green B (1997) Field energetics and water fluxes in marsupials. In: Saunders NR, Hinds LA, editors. Marsupial biology, recent research, new perspectives. Sydney: UNSW Press. pp. 143–162.

5. Nagy KA, Bradshaw SD (2000) Scaling of energy and water fluxes in free-living arid-zone Australian marsupials. Journal of Mammalogy 81: 962-970.

6. Smith AP, Nagy KA, Fleming MR, Green B (1982) Energy requirements and water turnover in free-living Leadbeater's possums, *Gymnobelideus leadbeateri* (Marsupialia: Petauridae). Australian Journal of Zoology 30: 737-749.

7. Bradshaw S, Morris K, Dickman C, Withers P, Murphy D (1994) Field metabolism and turnover in the Golden Bandicoot (*Isoodon auratus*) and other small mammals from Barrow Island, Western Australia. Australian Journal of Zoology 42: 29-41.

8. Nagy KA, Bradshaw SD, Clay BT (1991) Field metabolic rate, water flux, and food requirements of short- nosed bandicoots, *Isoodon obesulus* (Marsupiala: Peramelidae). Australian Journal of Zoology 39: 299-305.

9. Evans M, Green B, Newgrain K (2003) The field energetics and water fluxes of free-living wombats (Marsupialia: Vombatidae). Oecologia 137: 171-180.

10. Nagy K, Bradley A, Morris K (1989) Field metabolic rates, water fluxes, and feeding rates of quokkas, *Setonix brachyurus*, and tammars, *Macropus eugenii*, in Western Australia. Australian Journal of Zoology 37: 553-560.

11. Nagy K, Sanson G, Jacobsen N (1990) Comparative field energetics of two macropod marsupials and a ruminant. Wildlife Research 17: 591-599.

12. Munn AJ, Dawson TJ, McLeod SR, Croft DB, Thompson MB, et al. (2009) Field metabolic rate and water turnover of red kangaroos and sheep in an arid rangeland: An empirically derived dry-sheep-equivalent for kangaroos. Australian Journal of Zoology 57: 23-28.

13. Foley W, Kehl J, Nagy K, Kaplan I, Borsboom A (1990) Energy and water metabolism in free-living greater gliders, *Petauroides volans*. Australian Journal of Zoology 38: 1-9.

14. Quin DG, Riek A, Green S, Smith AP, Geiser F (2010) Seasonally constant field metabolic rates in free-ranging sugar gliders (*Petaurus breviceps*). Comparative Biochemistry and Physiology Part A: Molecular & Integrative Physiology 155: 336-340.

15. Green B, King D, Bradley A (1989) Water and energy metabolism and estimated food consumption rates of free-living Wambengers, *Phascogale calura* (Marsupialia, Dasyuridae). Wildlife Research 16: 501-507.

16. Krockenberger A (2003) Meeting the energy demands of reproduction in female koalas, *Phascolarctos cinereus*: evidence for energetic compensation. Journal of Comparative Physiology B-Biochemical Systemic and Environmental Physiology 173: 531-540.

17. Nagy K, Martin R (1985) Field metabolic rate, water flux, food consumption and time budget of Koalas, *Phascolarctos cinereus* (Marsupialia: Phascolarctidae) in Victoria. Australian Journal of Zoology 33: 655-665.

18. Munks SA, Green B (1995) Energy allocation for reproduction in a marsupial arboreal folivore, the common ringtail possum (*Pseudocheirus peregrinus*). Oecologia 101: 94-104.

19. Nagy K, Lee A, Martin R, Fleming M (1988) Field metabolic rate and food requirement of a small Dasyurid Marsupial, *Sminthopsis crassicaudata*. Australian Journal of Zoology 36: 293-299.

20. Nagy K, Meienberger C, Bradshaw S, Wooller R (1995) Field metabolic rate of a small marsupial mammal, the honey possum (*Tarsipes rostratus*). Journal of Mammalogy: 862-866.
